# Additional observations regarding glyphosate-based herbicides and developmental toxicity

**DOI:** 10.1073/pnas.2015865118

**Published:** 2020-12-22

**Authors:** William Reeves, S. Eliza Dunn

**Affiliations:** ^a^Regulatory Scientific Affairs, Regulatory Sciences, Bayer Crop Science, Chesterfield, MO 63017

Pu et al. (1) report that a soluble epoxide hydrolase (sEH) inhibitor mitigated the effects of maternal stress on F1 mice that exhibited atypical neurological symptoms. Research into the role of sEH in disease is valuable, and our comments do not cast doubt on the importance of sEH as a pharmaceutical target. We do have concerns with the conclusions regarding the cause of the observed outcomes—specifically, the conclusion that maternal exposure to high levels of a glyphosate-based herbicide played a direct role in the etiology of autism-like behaviors among offspring.

Pu et al. (1) induced maternal stress by administering high doses of Roundup Maxload herbicide through drinking water. We do not agree that the observed effects stem from consuming glyphosate, the herbicidal ingredient in Roundup Maxload. We reached this conclusion because the authors' primary sources do not support their hypothesis, the exposed mothers exhibited signs of malnutrition, and the toxicological properties of Roundup Maxload’s ingredients provide a more likely explanation.

Pu et al. (1) support their hypothesis with four citations, three of which (2–4) confuse correlation with causation by selectively citing or misinterpreting available literature. None of the disease associations conjectured by these three publications stems from empirical evidence, nor are they consistent with credible, reproducible toxicological data or observations in human populations. The fourth publication, after adjusting for other pesticides, shows no statistically significant association between proximity to glyphosate-treated agricultural fields during pregnancy and autism spectrum disorder diagnoses (5). None of the four citations supports the hypothesis that exposure to glyphosate or glyphosate-based herbicides during pregnancy causes autism or similar neurological symptoms in children.

In the study by Pu et al. (1), pregnant mice that consumed Roundup Maxload weighed 12 to 19% less than pregnant control mice. These differences indicate that adequate nutrition was not available for the exposed mothers. In humans, children of women who experienced famine during pregnancy had a twofold increased risk of schizophrenia (6) and antisocial personality disorder (7). Indeed, glyphosate is not neurologically, developmentally, or immunologically toxic (8), so it is unlikely that the observed outcomes were a direct effect of glyphosate ingestion. A more probable explanation is that pregnant mice in the Roundup Maxload-treated groups experienced famine-like conditions that caused neurological changes to their offspring.

In addition to glyphosate and water, Roundup Maxload contains a quaternary amine surfactant with the warning, “harmful if swallowed” (9). This quaternary amine may have made the water unpalatable or caused digestive tract irritation, making the animals less likely to eat or limiting nutrient absorption, possibly explaining the 20% mortality among offspring of exposed mothers. Additionally, glyphosate is not detectable in milk (8), so the blood monitoring data for offspring demonstrate direct consumption of Roundup Maxload, including the quaternary amine.

The sEH inhibitors mitigate pain and digestive tract ulcers (10). If Roundup Maxload or one of its constituents caused digestive tract irritation, it is possible that the sEH inhibitor mitigated these effects, allowing mothers receiving the sEH inhibitor to consume more food, thus mitigating effects on their offspring.

## Data Availability

The Bayer Agriculture BVBA, Safety data sheet is available in Figshare, https://doi.org/10.6084/m9.figshare.13335347.v1 (9).
